# TNFα Inhibits IGFBP-3 through Activation of p38α and Casein Kinase 2 in Human Retinal Endothelial Cells

**DOI:** 10.1371/journal.pone.0103578

**Published:** 2014-07-29

**Authors:** Qiuhua Zhang, Dylan Soderland, Jena J. Steinle

**Affiliations:** 1 Department of Ophthalmology, University of Tennessee Health Science Center, Memphis, Tennessee, United States of America; 2 Department of Anatomy and Neurobiology, University of Tennessee Health Science Center, Memphis, Tennessee, United States of America; 3 Department of Pharmaceutical Sciences, University of Tennessee Health Science Center, Memphis, Tennessee, United States of America; 4 Cell Systems Corp, Kirkland, Washington, United States of America; Queen's University Belfast, United Kingdom

## Abstract

We recently reported a reciprocal relationship between tumor necrosis factor alpha (TNFα) and insulin-like receptor growth factor binding protein 3 (IGFBP-3) in whole retina of normal and IGFBP-3 knockout mice. A similar relationship was also observed in cultured retinal endothelial cells (REC). We found that TNFα significantly reduced IGFBP-3 levels and vice-versa, IGFBP-3 can lower TNFα and TNFα receptor expression. Since IGFBP-3 is protective to the diabetic retina and TNFα is causative in the development of diabetic retinopathy, we wanted to better understand the cellular mechanisms by which TNFα can reduce IGFBP-3 levels. For these studies, primary human retinal endothelial cells (REC) were used since these cells undergo TNFα-mediated apoptosis under conditions of high glucose conditions and contribute to diabetic retinopathy. We first cultured REC in normal or high glucose, treated with exogenous TNFα, then measured changes in potential signaling pathways, with a focus on P38 mitogen-activated protein kinase alpha (P38α) and casein kinase 2 (CK2) as these pathways have been linked to both TNFα and IGFBP-3. We found that TNFα significantly increased phosphorylation of P38α and CK2. Furthermore, specific inhibitors of P38α or CK2 blocked TNFα inhibition of IGFBP-3 expression, demonstrating that TNFα reduces IGFBP-3 through activation of P38α and CK2. Since TNFα and IGFBP-3 are key mediators of retinal damage and protection respectively in diabetic retinopathy, increased understanding of the relationship between these two proteins will offer new therapeutic options for treatment.

## Introduction

Diabetic retinopathy is the leading cause of blindness, with numbers expected to reach epidemic levels by 2030. While a large number of factors are at play in this complicated disease, one that appears to have a major role is the presence of high levels of inflammation/apoptosis mediators such as TNFα [1]. In support of this hypothesis, TNFα knockout mice failed to develop diabetic retinopathy even when treated with a high galactose diet known to trigger manifestations of the disease [2]. Furthermore, TNFα has been suggested to be involved in inducing insulin resistance in adipocytes [3], [4], and retinal endothelial cells (REC) [5] through activation of insulin receptor substrate 1 (IRS-1)^Ser307^ and stimulation of apoptosis. Our work has suggested that additional TNFα pathways may also be involved in apoptosis related to retinopathy. For example, we have recently reported that TNFα can reduce insulin-like growth factor receptor binding protein 3 (IGFBP-3) levels in the retina, leading to increased apoptosis [6]. In the studies reported here, we further examine the link between TNFα and IGFBP-3 cascades and the ways in which they interact to create conditions of retinopathy.

The primary physiological function of IGFBP-3 is to deliver insulin-like growth factor 1 (IGF-1) to cells when it is required [7], [8]. However, more recent studies show that IGFBP-3 has additional actions independent of IGF-1 [9], [10]. Consistent with this notion, our *in vitro* findings in retinal endothelial cells indicate that high glucose reduces IGFBP-3 levels, which was correlated with increased TNFα levels [11]. We found similar results *in vivo*. IGFBP-3 KO mice had significantly increased retinal levels of TNFα [6]. Importantly, we have shown that intravitreal injections of IGFBP-3 into diabetic rat eye led to a significant reduction in TNFα [12]. In all cases, increases in IGFBP-3 are linked to decreases in TNFα; likewise, increases in TNFα are linked to decreases in IGFBP-3. The pathway by which TNFα inhibits IGFBP-3 is unknown and represents the focus of the current study. We know from previous studies that IGFBP-3 levels in retinal endothelial cells can be increased by PKA through activation of DNA-PK-induced phosphorylation of IGFBP-3 on serine 156 [13], and thus lead to reduced apoptosis. In the present study, we analyzed retinal endothelial cells grown in normal or high glucose to determine if TNFα inhibits IGFBP-3 by regulating IGFBP-3 phosphorylation. Our results show that a TNFα- p38α- CK2 pathway is activated under conditions of high glucose and results in a phosphorylation-mediated inhibition of IGFBP-3.

## Materials and Methods

### Reagents

Phospho-p38α MAPK, p38MAPK antibodies and SB202190 were purchased from Cell Signaling (Danvers, MA). Casein kinase 2 (CK2) antibody, human IGFBP-3 immunoassay ELISA kit, and TNFα were purchased from R & D (Minneapolis, MN). Phospho-CK2 antibody was purchased from Sigma-Aldrich (St. Louis, MO). BIRB 796 was purchased from Selleck (Houston, TX). Human IGFBP-3 siRNA and Non-Targeting siRNA #1 were purchased from Dharmacon RNAi Technologies (Chicago, IL). RNAimax was purchased from Invitrogen (Carlsbad, CA). SuperFect transfection reagent was bought from Qiagen (Valencia, CA). Horseradish peroxidase (HRP) conjugated secondary anti-rabbit antibodies purchased from Promega (Madison, WI). Enhanced chemiluminescence (ECL) for immunoblot development and signal detection was purchased from Amersham Biosciences (Piscataway, NJ, USA). TBB was purchased from Tocris Bioscience (Bristol, UK). IGFBP-3 NB plasmid DNA was a gift from Dr. Maria B. Grant (University of Florida).

### Cell Culture

Primary human REC were acquired from Cell System Corporation (CSC, Kirkland, Washington). Cells were grown in M131 medium containing microvascular growth supplements (Invitrogen) (MVGS), 10 µg/µl gentamycin and 0.25 µg/µl amphotericin B. For high glucose conditions, cells were transferred to high glucose (25 µM) (Cell Systems) medium, supplemented with MVGS and antibiotics for 3 days. Only primary cells within passages 6 were used. Cells were quiesced by incubating in high or normal glucose medium without growth factors for 24 hours and used to perform the experiments unless otherwise indicated.

### Mutagenesis

The IGFBP-3 NB plasmid DNA was a gift from Maria Grant, MD. Both the phosphorylation Serine^111^ site and the phosphorylation Serine^113^ site were mutated to Alanine to prevent their phosphorylation using the forward primer: 5′-CCAGGAAATGCTCGTGAGTCGGAGG-3′ and the reverse primer 5′-CCTCCGACTCACGAGCATTTCCTGG-3′ according to the QuikChange II XL Site-Directed Mutagenesis Kit manufacturer. The PCR sample reaction contained 5 µl of 10x reaction buffer, 10 ng dsDNA template, 125 ng of forward primer and 125 ng of reverse primer, 1 µl of dNTP mix, 3 µl QuikSolution, 1 µl of *pfuUltra* HF DNS polymerase and water to a total volume of 50 µl. PCR conditions used were initial heating at 95°C for 60 seconds to denature cDNA and activate the Taq DNA polymerase, followed by 18 cycles consisting of denaturation at 95°C for 50 seconds, annealing at 60°C for 50 seconds, and extension at 68°C for 300 seconds. After that, the amplification product was digested by DpnI and transformed by XL10-Gold ultracompetent cells. Colonies were selected, and the purified DNA was verified by DNA sequencing (University of Tennessee Health Science Center Molecular Science Core).

### Transfection of siRNA and plasmid DNA

ON-TARGETplus SMARTpool, human LRP-1 siRNA were purchased from Dharmacon, Inc. We used 4 sets of siRNA, with target sequences of GCUACAAAGUUGACUACGA, GAAAUGCUAGUGAGUCGGA, GCACAGAUACCCAGAACUU and GAAUAUGGUCCCUGCCGUA. siCONTROL Non-targeting siRNA #1 (Dharmacon) was used as a nonspecific control. REC were transfected with siRNA at a final concentration of 20 nM using RNAiMAX transfection reagent according to the manufacturer’s instructions. Briefly, 60 pmol of RNAi duplex was diluted in 150 µl of OPTI-MEM, and separately 9 µl of Lipofectamine RNAi/MAX was diluted in 150 µl of OPTI- MEM, each in an eppendorf tube. These two solutions were mixed and incubated for 10 min at room temperature. The transfection mixture was applied to REC cells in 60 mm dishes and incubated for 24 h at 37°C in a 5% CO_2_ incubator. The cells were used for experiments 24 hours after transfection. The cells were also transfected with IGFBP-3 NB plasmid DNA or its mutant at 1 µg/ml using SuperFect transfect reagent, according to the manufacturer’s instructions. Five µg of the plasmid was diluted with M131 medium to a total 150 µl. The transfection mixture was added to the REC in complete growth medium in a 60 mm dish. After 4 hours of incubation, REC were washed once with PBS, followed by fresh cell growth medium. The cells were used for experiment 24 hours after transfection.

### ELISA Analysis

An ELISA for IGFBP-3 levels was performed using an IGFBP-3 ELISA assay kit according to the manufacturer’s instructions to evaluate the IGFBP-3 levels following the treatments. Equal amount of protein was loaded onto the IGFBP-3 coated microplate. After washing away unbounded substances, an enzyme-linked polyclonal antibody specific for IGFBP-3 was added to the wells. A substrate solution was added to the wells and color developed in proportion to the amount of IGFBP-3 bound in the initial step. The intensity of the color was measured at 450 nm (vs. 540 nm reference).

*Western Blot Analysis-* After appropriate treatments and rinsing with cold phosphate-buffered saline, REC were scraped into lysis buffer containing protease and phosphatase inhibitors. Equal amounts of protein from the cell extracts were separated on the pre-cast tris-glycine gel (Invitrogen, Carlsbad, CA) and blotted onto a nitrocellulose membrane. After blocking in TBST (10 mM Tris-HCl buffer, pH 8.0, 150 mM NaCl, 0.1% Tween 20) containing 5% (w/v) BSA, the membrane was treated with phospho-p38α, p38, phospho-CK2 and CK2 antibodies followed by incubation with HRP conjugated secondary antibodies. The antigen-antibody complexes were detected using chemilluminescence reagent kit (Thermo Scientific).

### Statistics

All the experiments were repeated a minimum of three times, and the data are presented as mean ± SEM. Data was analyzed by Kruskal-Wallis test, following by Dunn’s testing with *p* values <0.05 were considered statistically significant. In the case of Western blotting, one representative blot is shown. The control was normalized to 1, and compared treatment to control based on fold change.

## Results

### TNFα significantly increased phosphorylation of P38α, leading to a decrease in IGFBP-3

**Figure 1:**
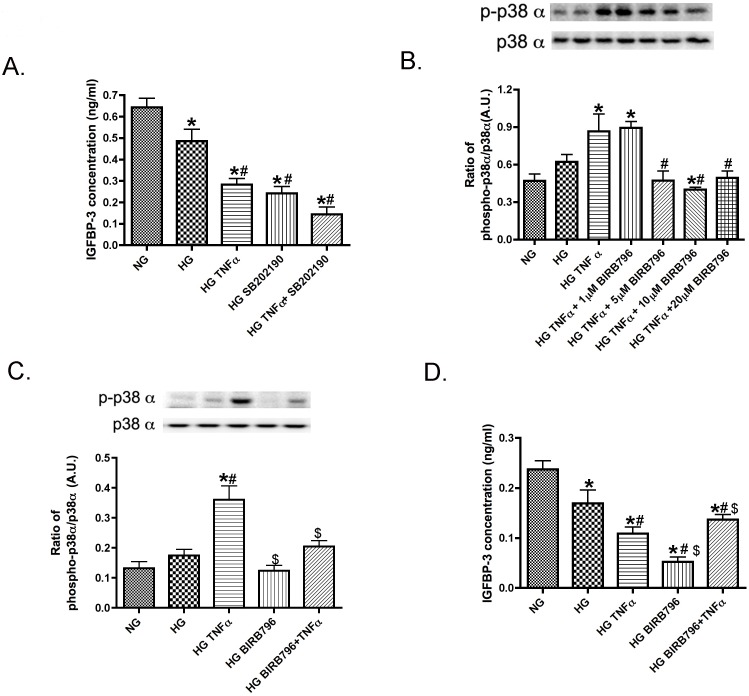
TNFα increases phosphorylation of p38α to regulate IGFBP-3 levels. Panel A presents ELISA results for IGFBP-3 following treatment with a non-selective p38 inhibitor (SB202190) and TNFα. Panel B shows Western Blot results of the ratio of phospho-p38α to total p38α with a dose-response curve for BIRB796, a p38α selective inhibitor. Panel C shows Western blot results for the ratio of phosphorylation of p38α to total p38 from REC grown in normal glucose (NG), high glucose (HG), high glucose + TNFα (HG+TNFα), high glucose + p38α inhibitor (HG+BIRB796), and high glucose + TNFα + p38α inhibitor (HG+TNFα+BIRB796). Panel D presents ELISA results for IGFBP-3 in cells treated with the same treatments. *P<0.05 vs. NG, #P<0.05 vs. HG, $P<0.05 vs. HG+TNFα. All data are mean ± SEM. Data is from 3–4 independent experiments for each treatment.

We have previously published that TNFα can significantly reduce IGFBP-3 levels in REC [6]. Others have reported that TNFα can regulate IGFBP-3 through activation of p38 in mammary epithelial cells [14]. To determine if p38 was involved in the TNFα regulation of IGFBP-3, we first grew REC in normal glucose (NG), high glucose (HG), or treated cells in HG with TNFα or a p38α inhibitor (SB202190 (non-selective) or BIRB796 (p38α selective)). We initiated this work using inhibition of all p38 isoforms using SB202190 and found the non-selective p38α and p38β inhibitor did not alter the role of TNFα in IGFBP-3 inhibition (Figure 1A). Since p38α is more commonly associated with inflammation and apoptosis, we then chose to use the p38α selective inhibitor, BIRB796. It is a highly selective inhibitor of p38α, with a Kd of 100 pM. BIRB796 can inhibit JNK2 but at 330-fold less than p38α. Because TNFα selectively activated p38α (Figure 1B), we next demonstrated that inhibition of p38α with BIRB796+TNFα treatment increased IGFBP-3 compared HG+TNFα only (Figure 1C & D). To verify we were using the best dose of BIRB796 in REC, we performed a dose-response curve for BIRB796 actions in inhibiting TNFα induced phospho-p38α activation and found BIRB796 had a role of p38α inhibition starting at 5 µM. To insure complete inhibition we used 10 µM BIRB796 for all subsequent experiments.

### TNFα-dependent phosphorylation of P38α stimulated casein kinase 2 (CK2) levels

**Figure 2:**
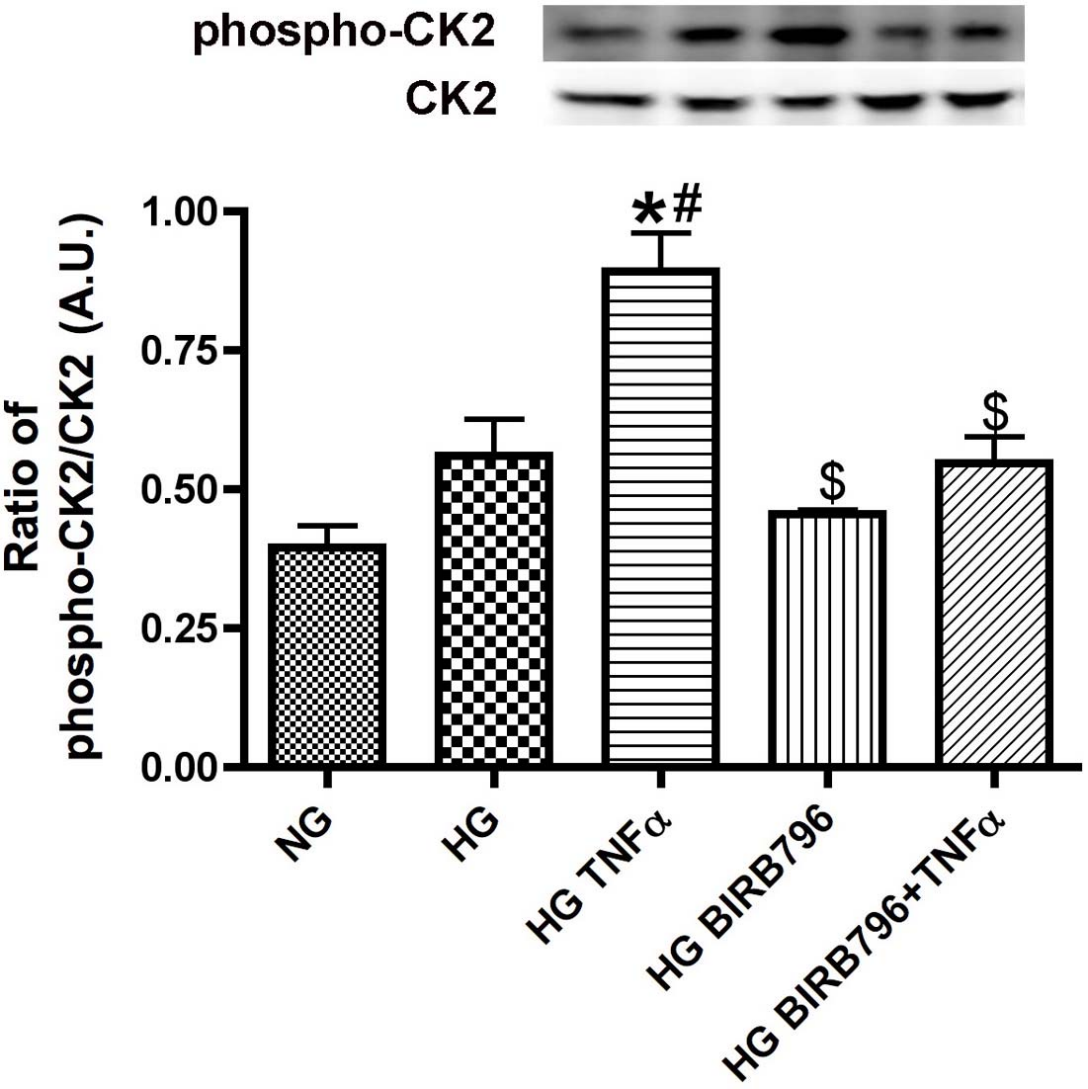
TNFα regulates CK2 levels through p38α. Western blot results for the ratio of phosphorylation of CK2 to total CK2 from REC grown in normal glucose (NG), high glucose (HG), high glucose + TNFα (HG+TNFα), high glucose + p38α inhibitor (HG+BIRB796), and high glucose + TNFα + p38α inhibitor (HG+TNFα+BIRB796). *P<0.05 vs. NG, #P<0.05 vs. HG, $P<0.05 vs. HG+TNFα. All data are mean ± SEM. Data is from 3–4 independent experiments for each treatment.

It has been shown that p38 can regulate CK2 levels in HeLa cells [15] and that CK2 can regulate IGFBP-3 in fibroblasts [16]. We wanted to determine if a similar p38 - CK2 pathway exists in REC and if so, whether it was regulated by TNFα. Figure 2 demonstrates that TNFα significantly increased CK2 phosphorylation, which was inhibited by pre-treatment with the selective P38α inhibitor, BIRB796. Taken with Figure 1 data, the data suggests that TNFα increased phosphorylation of both P38α and CK2 and that p38α regulated CK2 activity.

### CK2 inhibitor blocked TNFα inhibition of IGFBP-3 expression

**Figure 3:**
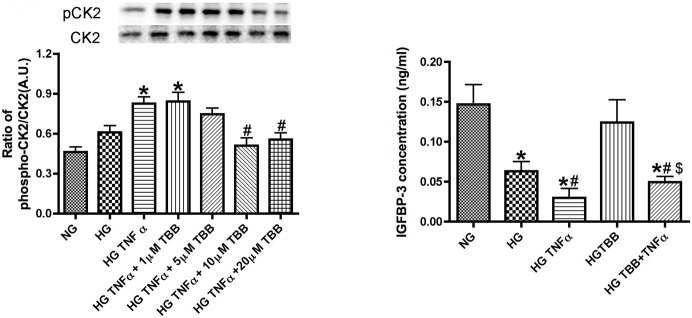
CK2 regulates IGFBP-3 levels. Left Panel is the Western Blot of the ratio of phospho-CK2 to total CK2 using different doses of TBB, a selective CK2 inhibitor, with TNFα treatment. Right panel is shows ELISA results for IGFBP-3 in REC treated with normal glucose (NG), high glucose (HG), high glucose + TNFα (HG+TNFα), high glucose + CK2 inhibitor (HG+TBB), and high glucose + TNFα + CK2 inhibitor (HG+TNFα+TBB). *P<0.05 vs. NG, #P<0.05 vs. HG, $P<0.05 vs. HG+TNFα. All data are mean ± SEM. Data is from 3–4 independent experiments for each treatment.

Because others have shown that CK2 regulates IGFBP-3 a transformed fibroblast cell line [16], we wanted to examine this pathway in REC. Figure 3 shows that 10 µM TBB inhibited phospho-CK2 significantly. TNFα significantly reduced IGFBP-3 levels, and this effect was blocked when the CK2 inhibitor (TBB) was combined with TNFα treatment (Figure 3). While TBB can potentially affect other pathways, it is a highly selective cell-permeable inhibitor of CK2 that functions through ATP/GTP-competitive actions. These data show that CK2 acts downstream from TNFα and is required for the subsequent inhibition of IGFBP-3.

### TNFα activation of P38α and CK2 led to inhibition of IGFBP-3 production through phosphorylation of IGFBP-3^Ser111/Ser113^

**Figure 4:**
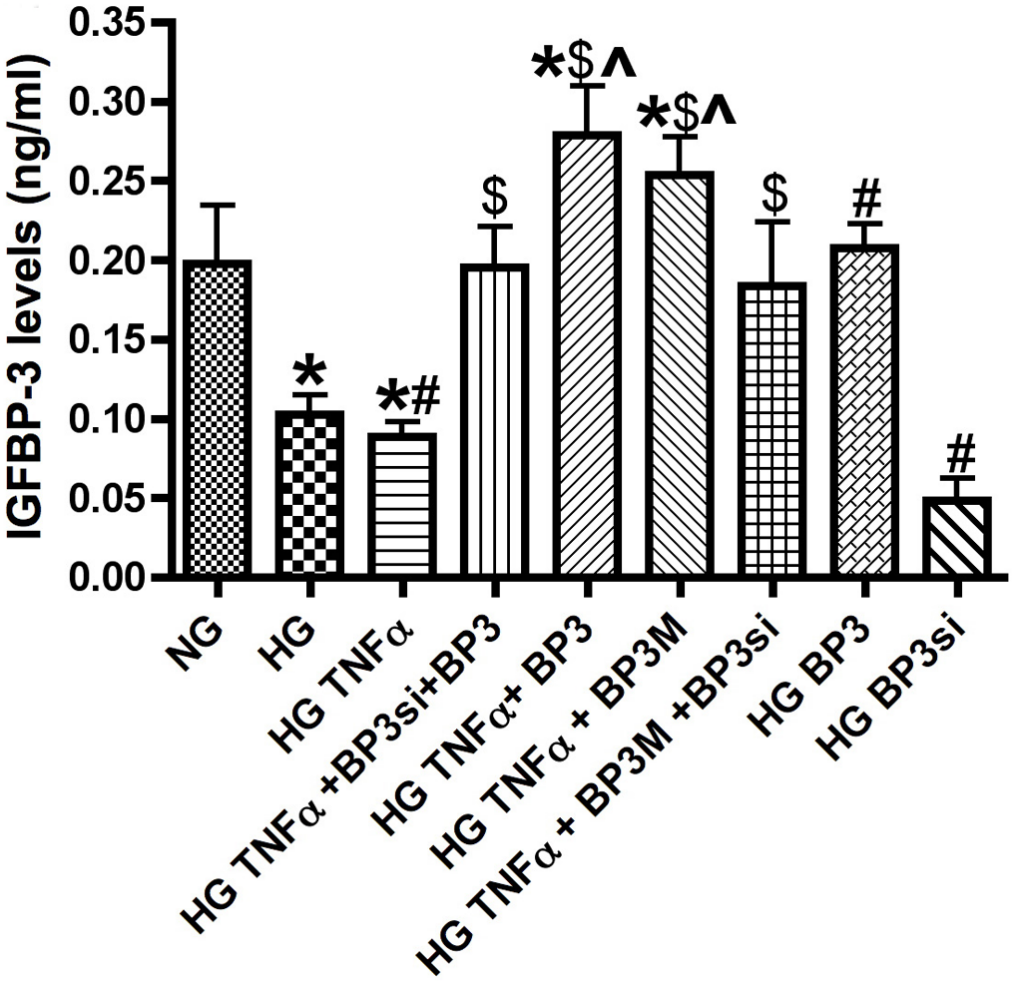
TNFα phosphorylates IGFBP-3 on Serine 111/113. IGFBP-3 ELISA results for REC treated with normal glucose (NG), high glucose (HG), high glucose + TNFα (HG+TNFα), high glucose + TNFα + IGFBP-3 mutant (mutation on Serine 111/113, (HG+TNFα+BP3^Ser-Ala^)), and high glucose + TNFα + IGFBP-3 siRNA + IGFBP-3 mutant (HG+TNFα+BP3si+BP3^Ser-Ala^). *P<0.05 vs. NG, #P<0.05 vs. HG, ∧P<0.05 vs. HG+TNFα $P<0.05 vs. HG+TNFα+BP3^Ser-Ala^. All data are mean ± SEM. Data is from 3–4 independent experiments for each treatment.

After demonstrating that TNFα increased phosphorylation of P38α and CK2, and ultimately caused a decrease in IGFBP-3 levels, our next step was to determine if specific phosphorylation sites on IGFBP-3 were targeted by CK2. We wanted to ascertain whether TNFα regulated IGFBP-3 levels through phosphorylation of Serine 111 and Serine 113 on IGFBP-3, as these are key sites for CK2 phosphorylation [17]. Unfortunately, no phospho-specific antibodies exist for these two sites. To answer our question without antibodies, we first performed site directed mutagenesis for Serine 111 and Serine 113 on IGFBP-3 NB plasmid to create IGFBP-3 NB^Ser-Ala^. We then wanted to verify that IGFBP-3 NB^Ser-Ala^ plasmid was able to increase total IGFBP-3 levels even in the presence of TNFα (Fig. 4), which include a control to verify successful reduction of IGFBP-3 with IGFBP-3 siRNA. As expected, levels of endogenous IGFBP-3 were decreased in response to high glucose and were further decreased when TNFα was added. In contrast, cells transfected with IGFBP-3 NB^Ser-Ala^ plasmid and treated with HG and TNFα had significantly higher levels of total IGFBP-3, which would be expected to include any remaining endogenous IGFBP-3 as well as mutated IGFBP-3 NB^Ser-Ala^. In order to distinguish between endogenous IGFBP-3 and mutated IGFBP-3 NB^Ser-Ala^, we pretreated cells with IGFBP-3 siRNA to block transcription of endogenous IGFBP-3. Under these conditions, levels of IGFBP-3 (presumably only IGFBP-3 NB^Ser-Ala^) remained significantly elevated even in the presence of TNFα, suggesting that the absence of active serine sites reduced the inhibitory effects of TNFα. Some may expect that transfection with the IGFBP-3 mutant would have reduced IGFBP-3 levels compared to wildtype IGFBP-3. However, we found that transfection with either the mutant or wildtype plasmid increased total IGFBP-3 levels. Others have reported this for other proteins [18]. The difference between mutant and wildtype IGFBP-3 levels would like be noted in the phospho-IGFBP-3 levels rather than total IGFBP-3 (measured here). Unfortunately, antibodies are not available to investigate this. Therefore, we then compared the HG+TNFa+BP3M with the HG+TNFa+BP3M+BP3siRNA, which should limit endogenous IGFBP-3 (wildtype IGFBP-3). When you compare these 2 bars, there is a significant difference. Therefore, we feel that these 2 data points suggest that TNFa does regulate IGFBP-3 through Serine111/113. Based on these findings, we conclude that TNFα acts on Serine 111 and Serine 113 phosphorylation sites to decrease IGFBP-3. A more direct test of this hypothesis will require production of site-specific antibodies.

## Discussion

### Reciprocal actions between TNFα and IGFBP-3

The search for key proteins involved in the retinal damage associated with diabetic retinopathy is an ongoing quest. In previous work using β-adrenergic receptor knockout mice [19], [20] or β-adrenergic receptor agonists [11], [21] under diabetic conditions, we found that TNFα is associated with both decreased β-adrenergic receptor signaling and diabetic-like changes. Importantly, TNFα knockout mice do not develop diabetes even when given a high galactose diet known to cause diabetes in control mice [1], [2]. While much is known about TNFα receptor downstream signaling in the pro-apoptosis pathway [22], we question whether TNFα may have broader effects on other pathways that indirectly or directly influence apoptosis. We have previously reported that another protein, IGFBP-3, is reduced in response to diabetes, in correlation with the observed increase in TNFα [11]. IGFBP-3 is of particular interest because it has a significant protective effect in retina. We wanted to determine if increased levels of TNFα could directly inhibit IGFBP-3 expression and thereby eliminate the protective actions of IGFBP-3 in the retina.

**Figure 5:**
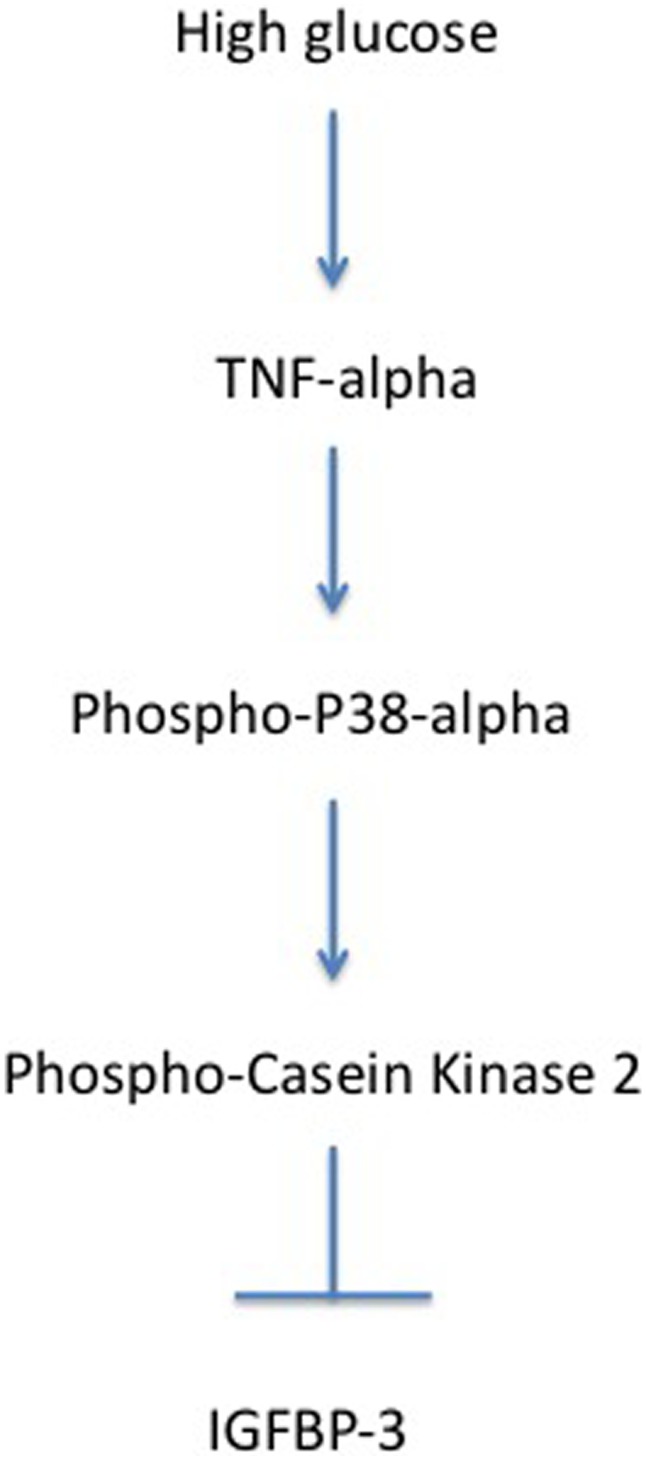
Schematic model illustrating how TNFα regulates IGFBP-3 through p38α and CK2.

Based on earlier studies by Coverley et al. [17], we examined whether TNFα could change IGFBP-3 levels by triggering the CK2-mediated serine phosphorylation of IGFBP-3. While TNFα can regulate CK2 itself, we found that TNFα preferentially phosphorylates p38α, leading to activation of CK2. The use of the p38α-specific inhibitor demonstrated that p38α is upstream of CK2. When p38α is inhibited, TNFα can no longer reduce IGFBP-3. These findings suggest that the pathway employed by TNFα to inhibit IGFBP-3 is TNFα→p38α→CK2-| IGFBP-3 (Figure 5).

### Possible role of TNFα and IGFBP-3 in insulin resistance

TNFα and IGFBP-3 may both be involved in insulin resistance, as we previously demonstrated that TNFα is key to activation of an insulin resistance phenotype in REC, noted by increased IRS-1^Ser307^ and IR^Tyr960^, with increased apoptosis of REC [5]. Additional studies demonstrate that treatment with IGFBP-3 restores normal insulin signal transduction in diabetic rats when administered by intravitreal injection [12]. We have also demonstrated that IGFBP-3 can regulate monocyte adhesion through altered TNFα actions [23]. Taken together, these findings of the interaction of TNFα and IGFBP-3 suggest that TNFα regulation of IGFBP-3 will alter insulin signaling in the diabetic retina. This will be a focus of future studies.

TNFα is a key player in diabetic retinopathy, as well as other ocular pathologies. In this study, we demonstrate the cellular signaling by which TNFα can regulate IGFBP-3. Since diabetes leads to increased TNFα and decreased IGFBP-3, increased understanding of the interrelationship of TNFα and IGFBP-3 will likely lead to refined therapies that can promote IGFBP-3 actions, while inhibiting TNFα activities. Since β-adrenergic receptor agonists appear to promote both of these pathways to protect the diabetic retina, reduction of TNFα to promote IGFBP-3 levels in the retina through β-adrenergic receptor stimulation may prove to be a new therapeutic option.
